# Age-Related Wayfinding Differences in Real Large-Scale Environments: Detrimental Motor Control Effects during Spatial Learning Are Mediated by Executive Decline?

**DOI:** 10.1371/journal.pone.0067193

**Published:** 2013-07-02

**Authors:** Mathieu Taillade, Hélène Sauzéon, Prashant Arvind Pala, Marie Déjos, Florian Larrue, Christian Gross, Bernard N’Kaoua

**Affiliations:** 1 Université Bordeaux, Handicap et système nerveux, EA 4136, Bordeaux, France; 2 INSERM, IFR Handicap, Handicap et système nerveux, EA 4136, Bordeaux, France; 3 Inria, Equipe Phoenix, Talence, France; 4 Inria, Equipe Potioc - LaBri, Talence, France; 5 Université Bordeaux, Institut des maladies neurodégénératives - UMR 5293, Bordeaux, France; University of California, San Francisco, United States of America

## Abstract

The aim of this study was to evaluate motor control activity (active vs. passive condition) with regards to wayfinding and spatial learning difficulties in large-scale spaces for older adults. We compared virtual reality (VR)-based wayfinding and spatial memory (survey and route knowledge) performances between 30 younger and 30 older adults. A significant effect of age was obtained on the wayfinding performances but not on the spatial memory performances. Specifically, the active condition deteriorated the survey measure in all of the participants and increased the age-related differences in the wayfinding performances. Importantly, the age-related differences in the wayfinding performances, after an active condition, were further mediated by the executive measures. All of the results relative to a detrimental effect of motor activity are discussed in terms of a dual task effect as well as executive decline associated with aging.

## Introduction

Large-scale spatial abilities are essential for everyday living activities [1]. Their decline with cognitive aging has been the scope of many recent studies, notably due to the development of Virtual Reality (VR) devices (for a review, see [2], [3]). Various cognitive factors have been identified as contributing to aging declines, such as the hippocampal-memory factors relative to spatial learning, but also the fronto-executive factors relative to path planning and execution (for a review, see [2], [3]). None of the previously published aging studies have specifically addressed the role of active motor exploration (compared to passive exposure) in age-related differences occurring in spatial behaviors in large-scale environments. Yet, in young adults, an active/passive effect is recognized as having an influence on navigation performances (for a review, see [4]). This issue is thus critical for VR-based aging studies, particularly when we consider that older adults have perceptual and sensorimotor difficulties (for a review, see [5]). Hence, the aim of this study was to evaluate the effect of motor control during VR-based spatial learning on the age-related differences in subsequent navigational and spatial memory performances, and then to study their connections to measures of executive and memory functioning.

Large-scale spatial skills, defined as the ability to learn and navigate in large spaces, have been the focus of numerous VR-based studies [4], [6], [7]. VR is particularly relevant to simulate realistic large-scale 3D spaces and, thus to overcome the lack of ecological validity of classical laboratory-based visuospatial tests [8], [9]. Spatial learning and wayfinding performances are probably the most representative of navigational behaviors in large-scale environments. Spatial learning can be defined as the process of spatial knowledge acquisition in large-scale spaces. Classical models propose a three-stage process (based on spatial knowledge levels) [10]: (1) the “landmark” (local or distal salient objects) level is acquired first, followed by (2) the “route” level (sequence of places and decisions) and, finally by (3) the “survey” knowledge level (also called configurational or directional knowledge). Originally described as sequential processing, recent findings suggest that survey knowledge can be acquired early, depending on the person’s sense of direction [11], [12], [13]. Many tasks address spatial knowledge acquisition, such as the picture classification task in which pictures must be arranged based on a given route (e.g., [14], [15]), landmark association and orientation decision tasks [15] for route level knowledge, the map drawing task (e.g., [14], [16]) or pointing tasks (direction and distance toward invisible targets; e.g., [17]) for survey level knowledge. According to Byrne, Becker and Burgess [18], the hippocampus and temporal lobe provide a long-term allocentric representation of space (i.e., also called survey representation), whereas the parietal lobes provide an egocentric representation of space.

Wayfinding is classically described as a purposeful, directed, and motivated movement from an origin to a specific distant destination, which cannot be directly perceived by the traveler [19], [20] and is likely dependent on executive functioning, such as planning abilities and decision making [1], [21]. Indeed, prefrontal cortex activations are found in relation with goal proximity [22], or with the planning and monitoring processes during navigation [7]. For this reason, wayfinding is seen as a strategic navigation behavior that requires the use of spatial knowledge in order to plan the correct path toward a specific target [20], [23]. Therefore, wayfinding is considered to be strongly dependent on both spatial memory and executive functioning [1], [3].

An age-related decline is widely reported in wayfinding as well as in spatial learning; this includes an age-related decline in landmark, route and survey knowledge acquisition in VR-based studies. However, this decline is mostly observed in maze-like environments, rather than in naturalistic large-scale spaces (for a review, see [2], [3]). Among the already published studies based on a realistic environment, most have investigated age-related differences in navigation tasks within a virtual environment (learning and recall phases in the VE [24], [25], [26], [27], [28], [29], while very few have investigated age differences in navigation tasks that require the use and transfer of spatial knowledge acquired in a VE to subsequently perform a navigation task under real conditions [21], [30], [31]. Interestingly, studies using transfer tasks reported an age-related effect on the wayfinding performances [21], [30], [31], but failed to find this effect on the spatial memory performances (route and survey knowledge measures) [21], [30]. Taillade et al. [21] and Foreman et al. [30] showed differences on wayfinding performances between younger and older adults but no difference for spatial memory tasks. Foreman et al. [30] studied the transfer of spatial knowledge from a virtual shopping mall to its real version. Taillade et al. [21] used a much larger VE than the former study, including measures assessing the efficiency of the planned routes in a wayfinding task (direction errors observed and stops to choose the directions). Additionally, they reported that the wayfinding difficulties experienced by the older adults were strongly related to their declines in the executive and spatial memory functioning measures (assessed by conventional paper-pencil tests), whereas the wayfinding performances of the younger adults were only mediated by the executive measures. Based on these results, these authors proposed that the spared spatial memory performances in the elderly adults are the result of a benefit arising from a re-test learning effect due either to direct re-exposure to the environment during the wayfinding task or by indirect re-exposure during the memory tasks (picture classification and map drawing task). This assumption was supported by the association of a decline in the wayfinding task with both a memory and executive decline, suggesting that the elderly adults completed their spatial learning during the transfer task. In other words, during this task, the older adults carried out path planning or navigation monitoring (path progression, spatial updating, etc.) in the real condition by prolonging the spatial information encoding phase, thus rendering the task more difficult for them. When considered together, the elderly subjects performed as well as the younger subjects on the spatial memory tasks, but did not perform as well on the wayfinding task.

An important issue that is often overlooked in VR-based aging studies is the role played by the motor activities engaged during the spatial learning phase for controlling the VR interfaces. The use of a motor interface, such as a mouse or a joystick (or even a steering wheel for a driving simulator), have long been used as a tool to enhance spatial learning or wayfinding performances (for review, see [4]). According to the distinction between physical vs. cognitive (referring to attention, decision making and mental manipulation, according to Chrastil and Warren’s proposal [4] activity during environment exploration by Wilson et al. [32] the specific capture of motor activities can refer to comparisons between the two VR exploration conditions: (1) motor passive navigation (the subject visualizes a pre-recorded route in a virtual environment without moving or making decisions), and (2) motor active navigation (the subject moves using a motor interface while following directions given by an experimenter in order to follow a given route). This comparison is usually labeled *active exploration or navigation effect* (for a review, see [4]).

For the younger adults, the active conditions had a positive effect on the wayfinding performance based on a virtual-real transfer task [14], [33], [34], [35], whereas contradictory results were found for the spatial learning performances relative to the different levels of spatial knowledge (landmark, route or survey) (e.g., *for a positive effect,* see [36], [37], [38], *for no effect*, see [39], [40], [41], [42], [43], *for a negative effect*, see: [44]). Accordingly, it could be expected that active exploration would have a positive effect on the wayfinding performances of the younger adults, whereas it is more difficult to determine the effect with regards to the three-levels of spatial knowledge. As highlighted by Chrastil and Warren [4], the inconsistent results concerning the active-passive effect might be due to a discrepancy in the experimental design, notably with regards to the manipulation of the motor activity (e.g., treadmill, steering wheel, joystick, all differing in terms of sensorimotor stimulation) and its confounding effects with psychological activity (decision making, attention and mental manipulation).

Regarding the aging effect, to the best of our knowledge, no study has directly addressed the age-related difference in VR-based wayfinding tasks with respect to an active/passive exploration manipulation. This is surprising since motor control is critical for VR aging study designs. In fact, only the study by Plancher et al. [45], using a driving simulator, assessed the role of active (driver) and passive (passenger) learning in age-related differences on episodic memory measures (details relative to objects, and spatial information such as the position of the objects relative to the body and along the route, and direction changes relative to the objects). They found no significant effect of the “driving” vs. “passenger” conditions on the memory performances. The lack of benefit is interpreted as a dual task effect during the encoding phase in which participants do not have enough resources in the active condition to pay attention to every detail in the environment. A similar explanation is also advanced in other aging studies testing the possible negative effect of sensorimotor control on spatial memory performances: Lövdén et al. [28] found that walking on a treadmill without any help to maintain balance had a negative effect on the spatial memory performances of the older participants in a virtual museum, but not for the younger participants. The authors concluded that the sensorimotor control required for the motor activities could place the participants in a dual-task situation, for which robust age-related difficulties are usually reported in a multi-task condition, involving simultaneous walking and cognitive activities (e.g., [46], [47], [48] for example). According to the sensory deficit theory of aging, age-related deficits in sensory processing play a major role in age-related cognitive decline ([49], for a review, see [5]). In this vein, older adults may tend to “prioritize” sensorimotor control (requiring working memory resources) over cognitive control when dual “cognitive-sensorimotor” control is necessary [47], [50]. This phenomenon, in addition to executive memory decline, could also contribute to the general decline in the spatial memory and wayfinding performances, particularly in light of an aging decline in sensorimotor information processing, including difficulties in optic flow processing [51], [52], path integration processes [53] and motor control [54]. Note that this dual task effect could be different between route and survey acquisition since the acquisition of route knowledge is more automatic than the acquisition of survey knowledge [4].

Hence, the aim of this study was to assess age-related differences in terms of “active-passive” motor exploration in spatial memory and wayfinding performances in a large district of the city of Bordeaux, and to study their connections with the mediating role of age-related declines in spatial, memory, executive abilities. According to the sensory deficit hypothesis, we expected that sensorimotor control in the active condition is more costly in terms of cognitive resources for elderly adults than in passive condition.

## Methods

### Participants

Thirty younger adults (mean age = 23.12; *SD = *2.97; age range: 18–30) and thirty older adults (mean age = 64.50; *SD = *3.68; age range: 58–72) participated in this study. The inclusion criteria for both the younger and older adults were the absence of foreknowledge of the district, being right handed and to have no past or present neurologic disorders. Also, all of the participants were volunteers and native French speakers. From a general questionnaire, they reported they were healthy and without any visual, neurological or psychiatric disorders. This self-assessment procedure, although possibly limitative, was designed so as to not lengthen the experiment duration (which was already long with the virtual experiment and the neuro-cognitive assessment). The younger adults were recruited at the University of Bordeaux and the older adults were recruited from a Senior University in Bordeaux (“Université du Temps Libre”). In addition, the inclusion criteria for the older participants also included not having a global cognitive deficiency and therefore, the older subjects were tested with the Mattis Dementia Rating Scale as an exclusion test (exclusion for a score <129).

Even if this study does not meet the criteria for the CPP-III assessment, each participant signed a consent form in order to obtain the approval of each participant, as recommended by the CPP-III and the Helsinki convention. This document explains the process and the reasons for the study and how the behavioral data collected will be used. A written informed consent was obtained from each participant. All data were analyzed anonymously.

The younger and elderly groups were divided between “active” and “passive” groups. All of the subjects had to complete a French version of the Simulator Sickness Questionnaire (SSQ; [55]) immediately after the learning phase in the VE. This questionnaire measures the severity of sickness induced by 3D simulators. The questionnaire consisted of a list of 16 symptoms clustered under three factors: Oculomotor, Nausea and Dizziness. Symptoms were associated with presence*/*absence and with their degree of severity. Answers were scored 0 for “absence” of symptoms, 1 for “mild” symptoms, 2 for “present” and 3 for “severe”. The weighted totals of the scale scores and conversion formulas are the same as those used by Kennedy et al. [55]. Participants also had to rate their New Technology (NTIC) experience with computers and computer games ([56]). This includes three items that were rated from 0 to 7; the maximum score was 21 for each participant. The Everyday Visuospatial Difficulties (EVSD) survey along with the Spatial Orientation questionnaire (wayfinding and object memory subscores; [57]) and the French adaptation of the Mill Hill Vocabulary Scale [58] were also administered.

There were no significant inter-group differences between the active and passive conditions for age (p>.600), education level (p>.200), the Mill Hill Vocabulary test (p>.800), the Skelton Wayfinding score (p>.900), the Skelton Object Memory score (p>.900), the NTIC (p>.200) and the SSQ (p>.400) scores. There was no interaction between the age and navigation (active vs. passive) conditions with regards to the education level (p>.200), the Mill Hill Vocabulary test (p>.800), the Skelton Wayfinding score (p>.900), the Skelton Object Memory score (p>.900), the NTIC (p>.200) and the SSQ (p>.400) scores.

There was no age effect for education level (p>.400) or the Skelton Object Memory score (p>.200). There was a significant age effect for the Mill Hill Vocabulary test (p<.0001), the Skelton Wayfinding score (p<.05), and the NTIC (p<.0001). The younger adults had higher scores on the Skelton Wayfinding questionnaire, indicating that they report more wayfinding difficulties than the older adults, in agreement with our previous experiment (Taillade et al., 2012). The older adults had higher scores on the Mill Hill Vocabulary test and had lower scores on the NTIC questionnaire.

The characteristics of the subjects for each group are presented in Table 1.

**Table 1:** Characteristics of the participants.

	Younger adults		Older adults			
	Active mean (SD)	Passive mean (SD)	Active mean (SD)	Passive mean (SD)	Group effects	
N	15	15	15	15		
Age	23.13 (3.13)	23.20 (2.96)	64.87 (2.90)	64.07 (4.11)		F(1,58) = 2330.48; p<.0001
Education Level	15.33 (2.09)	15.67 (1.72)	15.87 (3.02)	14.20 (2.43)		F(1,58) = 1.194; p>.200
Mill Hill Vocabulary	23.40 (4.29)	23.80 (4.84)	28.47(2.33)	28.40 (3.20)		F(1,58) = 24.367; p<.0001
Skelton Wayfinding	27.20 (13.95)	27.33 (13.48)	19.33 (12.42)	20.00 (8.77)		F(1,58) = 5.703; p<.05
Skelton Object Memory	18.20 (7.04)	17.67 (6.68)	19.87 (8.12)	20.26 (5.03)		F(1,58) = 1.471; p>.200
NTIC	15.00 (4.63)	13.31 (4.77)	9.40 (4.00)	8.47 (2.29)		F(1,58) = 25.239; p<.0001
SSQ	83.32 (100.05)	219.73 (238.31)	194.70 (271.31)	143.72 (154.23)		F(1,58) = 0.084; p>.700

### VR-based Material and Procedure

All of the material and procedures are derived from previous studies demonstrating the relevance of VR-based applications to study spatial learning [14], [21], [33], [34], [35], [59]. The VE was a replica of the district near the Bordeaux hospital. It was created using the Virtools© software. Significant landmarks (signposts, signs, and urban furniture) were included in the VE. The apparatus used in the VR room was a Dell© personal computer (3 GHz, 5 Gb RAM) with a NVIDIA© Quadro FX 4400 graphics card, a F1+ © projector, a 2×1.88 meter screen. In addition, in order to actively explore the VE, a Thrustmaster joystick was programmed with three degrees of liberty for displacements: forward translational movements (push); (2) angular rotations to the left or (3) to the right (to lean left or right in a 45° angle).

**Figure 1:**
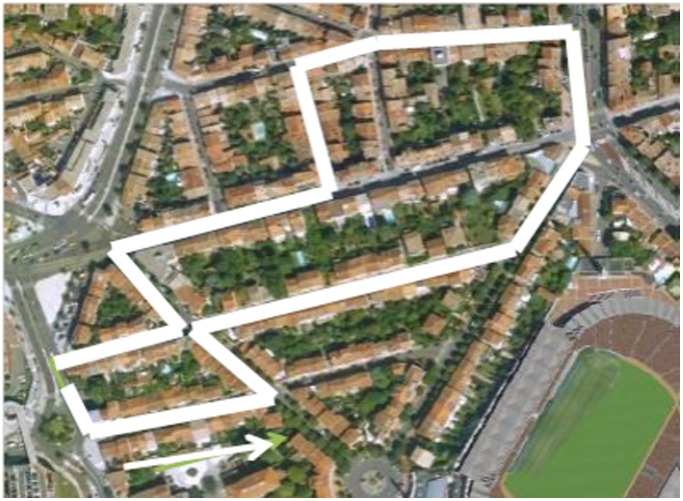
Representation of the district area and of the route used for the experiment.

The procedure was divided into three steps as follows: (1) *a training phase* (15 minutes), where each participant was trained to navigate in an unused part of the VE, to allow the participants to be familiarized with the virtual navigation and joystick use and to confirm that none of the participants had major simulator sickness (mostly for the older adults); more precisely, during the training phase, they had to control their trajectory (linear and rotations) on a street and between trees until they made no translational or rotational mistakes; (2) *a learning phase* (15 minutes), where the participants learned a route in the VE, which was 787 meters long, and composed of nine streets, 13 intersections and 11 direction changes (Figure 1); (3) and *a restitution phase*, the participants performed after a 10-minute retention interval two kinds of tasks as follows: wayfinding task and spatial memory tasks.

The participants used a joystick to control their movements in the VE and carried out instructions given by the experimenter in the “active” condition. In the “passive” condition, the participants did not interact with the VE and only had to watch the screen displaying the travel (as in our previous studies [14], [33], [34], [35]). The same verbal instructions were given in both the active and passive conditions.

#### Wayfinding task

The participants were asked to replicate the path they learned in the VE in the real Bordeaux district. To this end, the participants were brought to the starting point in the real district, which corresponded to the virtual one, and were asked to recall the path. Wrong turns and stops before deciding to change directions (when the subject stops more than five seconds and looks around) were counted. When a mistake occurred after a stop, both were counted. If the subject made a wrong decision, he was shown the correct direction by the experimenter and was allowed to continue on his/her way. Thus, two scores were calculated from this task: the number of direction errors (wrong turns) and the number of stops during the wayfinding task, as a probe of the use of spatial representation to perform a navigational task [14], [21], [33], [34], [35], [60].

#### Spatial memory tasks

The participants carried out two tasks. The first was a *map drawing* task, participants are known to have strong performances when they have developed a good spatial cognitive map of the environment [14], [33], [34], [35], [59], [60]: the subject has to draw the route learned in the VE on a blank sheet of paper. The drawing had to be made of connected segments, representing the linear locomotion and direction changes. To help the participants, an arrow was provided on a blank sheet of paper to show the starting point. Then, the participants were asked to draw starting from this position, the configurational sketch of the path by way of an outline made up of connected segments. The score included two criteria: the presence of a loop and the number of correct directions given from the beginning of the path. The maximum possible score is 11. The second task was a *picture classification* task, participants are known to have strong performances when they have well-developed route knowledge of the performed path [14], [33], [34], [35], [60]. The goal of this task was to chronologically order 12 pictures corresponding to different points of views of the district encountered along the route. The score is a sequence score: 1 point is given if the picture position corresponds to the correct position in the overall sequence and half of a point is given if the position is incorrect but near a picture that immediately follows with respect to the chronological order. The maximum possible score is 12. From the above descriptions, the picture classification and map drawing tasks were used as a probe of “route” and “survey” representations, respectively. The order of the tasks was counterbalanced between the subjects.

### Neuro-cognitive Assessment

Several neuropsychological tests were administered to each participant to estimate their cognitive functioning, assessing three cognitive domains: visuospatial (VS) abilities, visuospatial memory (VS-M), and executive functioning (EF). These tests were administered before and after the training sessions and the learning test in the VR, and their order was counterbalanced between the subjects. Composite scores relative to each one of three domains were calculated.

#### Visuospatial (VS) functioning included

(1) Mental rotation abilities that are probed with the Mental Rotation Test (MRT, by Vandenberg and Kuse [61]); this test requires participants to mentally rotate 3-dimensional figures in order to make a similarity judgment between them; (2) visuospatial working memory, which is probed with the Backward Corsi Span Test (BCS) as part of the WMS-III (Wechsler Memory Scale-III; [62]); this test requires participants to recall, in reverse order, the sequence of squares to which the experimenter is pointing. These squares are positioned on a “tray”.

#### Visuospatial Memory (VS-M) functioning included

*(1)* the immediate free recall and (2) the delayed recognition performances from the Visual Reproduction Test (WMS-III; [62]). Participants taking the immediate recall test must draw five geometrical figures after a retention interval of 10 seconds. For the delayed recognition test, after a retention interval of 20 minutes, the participants must make Yes/No recognition decisions for the figures presented (previously studied figures mixed with unstudied figures).

#### Executive Functioning (EF) included

(1) cognitive flexibility with part B of the Trail Making Test (TMT B; [63]) and (2) inductive reasoning abilities with the Raven’s Matrices Test (RMT standard form; [64]). Participants taking part B of the TMT must draw a line in order to connect letters and numbers in alphabetic order and in increasing order, respectively (letters and numbers are alternated). The RMT is composed of sixty problems with growing difficulty in terms of inductive inferences. The problems require the participants to choose between six or eight figures that can be used to complete a figure or a series of geometrical figures. The participants have 25 minutes to complete the problems.

## Results

The results are organized into three parts. The first refers to the VR-based results in the younger and the older adults from the active/passive exploration manipulation within the virtual environment. The second presents the age-related differences that occurred in the three neuro-cognitive domains (VS; VS-M; EF) assessed with conventional paper-pencil tests. And finally, the third focuses on the correlation results between the VR-based performances and the neurocognitive measures expressed by composite *z* scores.

### VR-Based Performances

Two-way ANCOVA [2 (age group: younger adults; older adults) x 2 (learning condition: active; passive)] analyses were carried out on each spatial learning and navigation measure using the NTIC score as a covariate variable. As no significant mediating effect for the NTIC variable was observed (p>.05) for each of the studied dependant measures, two-way ANOVA [2 (age group: younger adults; older adults) x 2 (learning condition: active; passive)] analyses were carried out.

#### Wayfinding task

**Figure 2:**
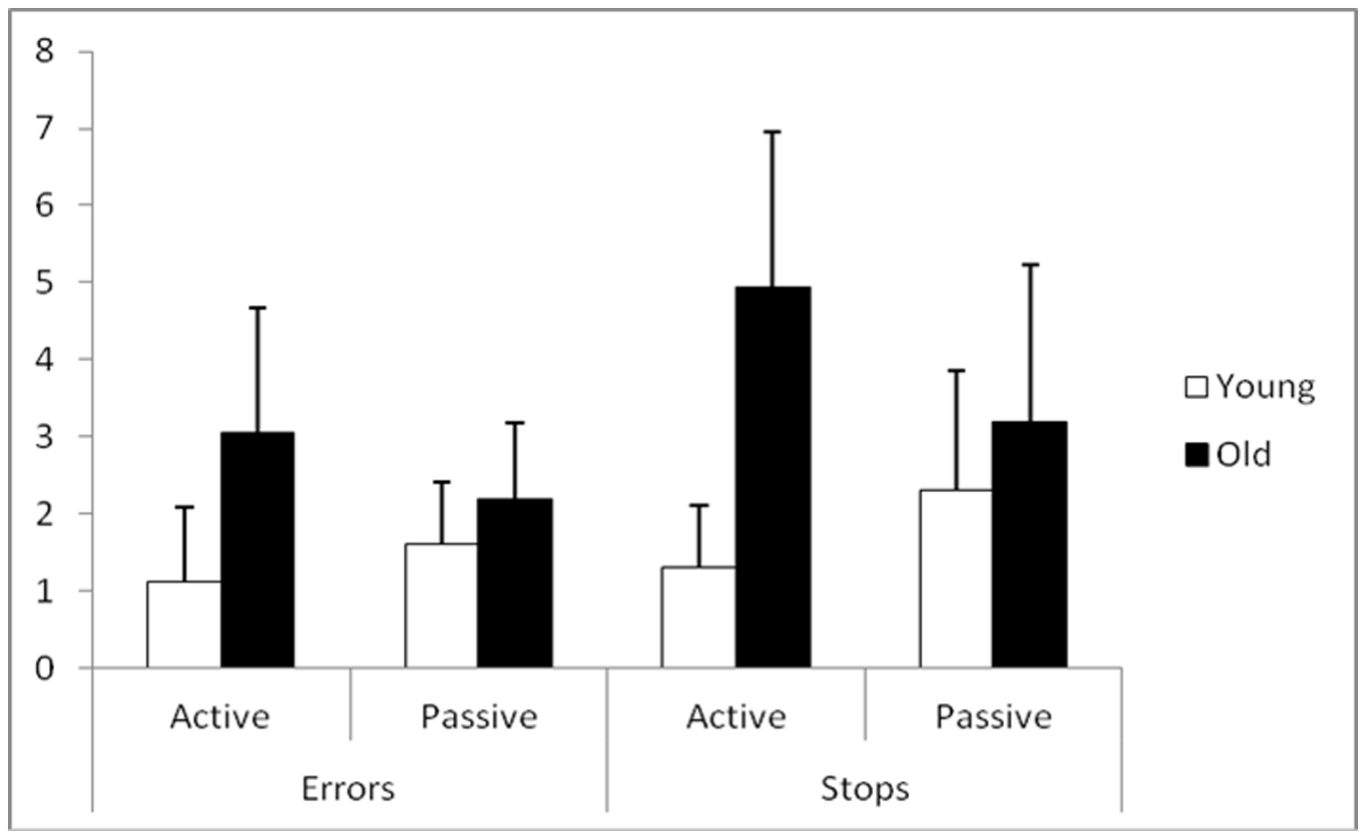
Wayfinding performances as a function of age and the navigation condition. Mean and Standard deviation for the number of Wayfinding errors and stops, according to the active and passive learning conditions and age; ANOVA (Group x Exploration) results.

For the error scores, the results showed a significant effect of age [F(1,56) = 19.512; p<.0001]: the younger participants made fewer errors than the older adults. No effect of learning condition [F(1,56) = 0.439; p>.500] was obtained; however, the interaction between age and learning condition was significant [F(1,56) = 7.563; p<.05]: the active participants in the younger group made fewer errors whereas more errors were made in the older group. Age differences were higher in the active condition (p<.0002) compared to the passive condition (p<.03) (Figure 2).

For the stop scores, a significant effect of age [F(1,56) = 28.886; p<.0001] was found where the younger participants made fewer stops than the older adults. Also, only the interaction between age and learning condition [F(1,56) = 10.788; p<.01] was significant: the active participants made fewer stops in the young grouper but more stops in the older group. Age differences were higher in the active condition (p<.0001) compared to the passive condition (p<.03) (Figure 2).

#### Spatial memory tasks

**Figure 3:**
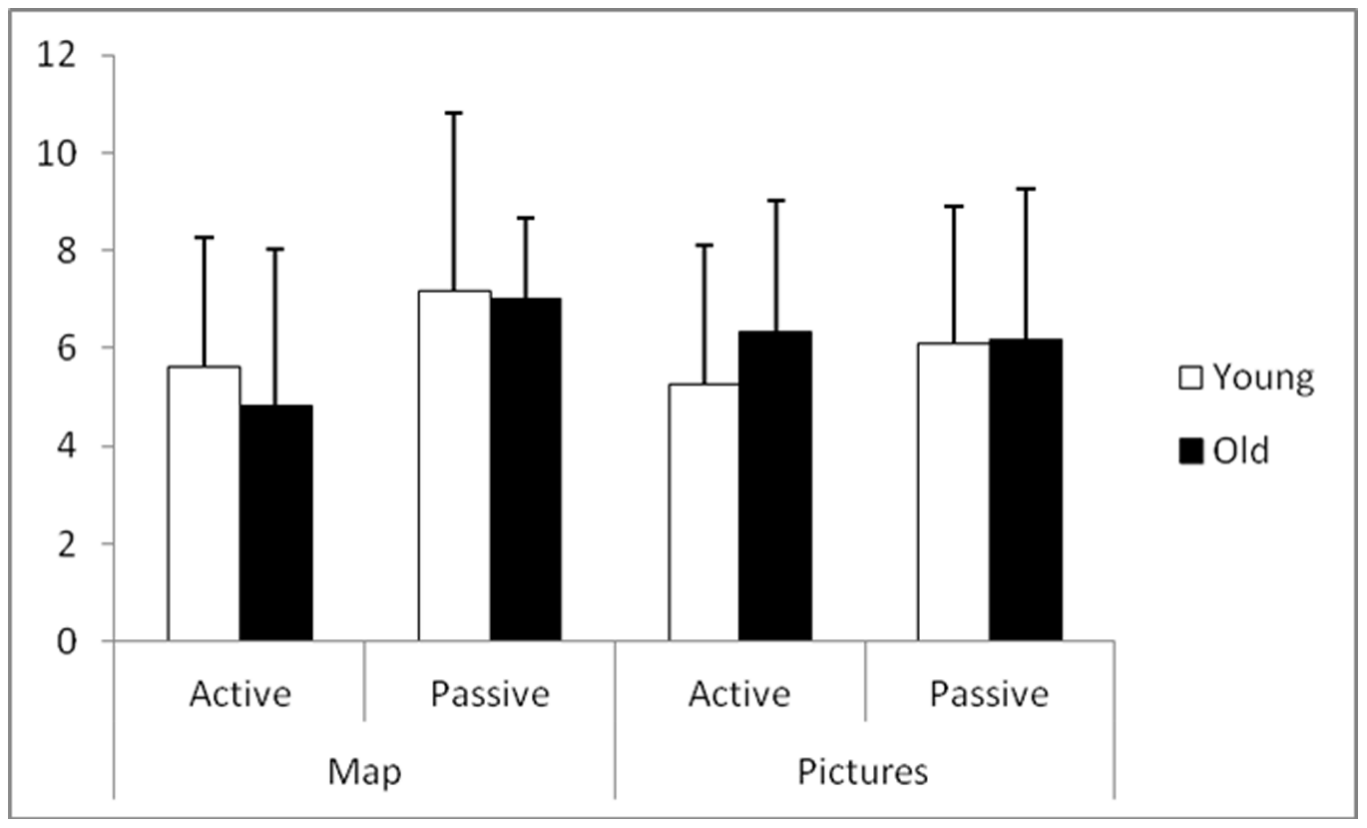
Spatial memory performances as a function of age and the navigation condition. Mean and Standard-deviation for the sketch drawing scores and the picture classification scores, according to the active and passive learning conditions and age; ANOVA (Group x Exploration) results.

For the map task, the results showed no significant effect of age (F(1,56) = 0.479; p>.400), a significant effect of navigation mode (F(1,56) = 6.742; p<.05) and no significant interaction between them (F (1,56) = 0.187; p>.600) (Figure 3).

For the picture classification task, the results showed no significant effect of age (F(1,56) = 0.692; p>.400) and navigation mode (F(1,56) = 0.232; p>.600), and no significant interaction between these two factors (F(1,56) = 0.49; p>.400) (Figure 3).

### Neuro-cognitive Assessment

Two-way ANOVA [2 (age group: younger adults; older adults) x 2 (active-passive condition: active; passive)] analyses were carried out on each neuro-cognitive measure.

All of these analyses indicated no simple or interaction effect, including the active-passive condition factor. By contrast, an age effect was significantly observed in all of the dependant measures relative to the three cognitive domains of interest for the navigation behaviors. The younger participants performed better than their older counterparts with regards to the visuospatial abilities (VS), visuospatial memory (VSM) and executive functioning (EF) measures (Table 2).

**Table 2:** Participant’s scores on the neuropsychological assessments and the ANOVA (Group x Exploration) results.

	YOUNGER GROUP		OLDER GROUP		ANOVA (Group×Exploration) *significant effects*
Navigation mode	Active mean (SD)	Passive mean (SD)	Active mean (SD)	Passive mean (SD)	
**VisuoSpatialabilities**					group effect:
Mental Rotation Test	8.81 (1.42)	8.56 (2.12)	7.37 (1.50)	6.25 (1.78)	F(1,56) = 22.443; p<.0001
BCS	19.12 (9.20)	17.81 (8.50)	10.37 (5.94)	9.19 (4.79)	F(1,56) = 18.842; p<.0001
**Visuospatial Memory**					group effect:
Mem III IR	95.25 (8.62)	98.56 (5.09)	87.19 (12.38)	85.12 (9.13)	F(1,56) = 25.212; p<.0001
Mem III Rec	46.37 (1.74)	46.69 (0.87)	43.44 (3.48)	43.50 (2.80)	F(1, 56) = 13.489; p<.001
**Executivefunctioning**					group effect:
TMT B	45.62 (16.66)	46.41 (15.70)	64.44 (22.57)	62.91 (24.20)	F(1,56) = 12.290; p<.001
Raven’s Matrices Test	55.87 (2.87)	54.44 (3.78)	47.37 (4.32)	48.56 (5.02)	F(1,56) = 49.824; p<.0001

### Correlations between the Wayfinding Performances and the Neuro-cognitive Measures

To analyze the relationship between VR-based wayfinding and the spatial memory performances, on the one hand, and neurocognitive measures, on the other, according to the learning conditions (active vs. passive), we constructed composite z scores for the wayfinding and map drawing performances and for the three cognitive domains considered in our experiment. For the two wayfinding scores, a z score was calculated from the errors and stop performances to analyze the correlations between the wayfinding performances and the neuro-cognitive measures. The VS functioning z score was constructed with the mental rotation and BCS tests; the VS-M z score was constructed with the immediate free recall and the delayed recognition task of the WMS-III; and finally, the EF z score was constructed with the Raven’s matrices and the trail making test. We calculated the correlations between age and the wayfinding z score, partialled out for each one of z scores for the neurocognitive indices. A comparison of the correlations before and after each partialling out was also done in the active and passive conditions according to Fisher’s transformation procedure (with the limit values for Z at 1.96).

As indicated in Table 3, the wayfinding z score in the active condition was significantly correlated with age (r = .72; p<.0001). This correlation, partialled out for VS, VS-M and EF z scores, remained significant: r = .58, (p<.001); r = .63 (p<.0001); and r = .46 (p<.01), respectively. However, the correlation comparisons indicated that, when controlled by the EF z score variable, the r value between age and the wayfinding score was significantly modified.

**Table 3:** Correlations between the wayfinding z scores and age (the p values are in parentheses) for the active and passive conditions, followed by the same correlations partialled out for each one of the neurocognitive z scores (VS-M z, VS-F z and EFz).

		*Partialled out for*		
	z Wfg * Age	VS-F	VS-M	EF
**Active**	.72(<.0001)	.58(<.001)	.63(<.0001)	.46(<.05)
**Z**		1.24	0.85	**2.01**
**Passive**	.47(<.001)	.15(>.400)	.16(>.300)	.36(>.09)
**Z**		1.56	1.52	0.61

The Z values are calculated for correlation comparisons.

In the passive condition, the wayfinding z score was significantly correlated with age (r = .47, p<.001). This correlation, partialled out for VS, VS-M and EF z scores, was no longer significant: r = .15, (p>.400); r = .16, (p>.300); and r = .36, (p>.05), respectively. However, these changes with respect to the r value did not reach significance.

## Discussion

To date, no study has assessed age-related differences in navigation and spatial learning tasks according to active vs. passive motor exploration manipulations. Additionally, no study has addressed the issue of age differences in the active exploration effect, in light of the mediating effect of aging decline in spatial abilities, memory or executive functioning. The results presented here provide insights on these issues.

Among the main results reported here, the first set of results show a negative effect of age on the wayfinding performances, but no negative effect of age on the spatial memory performances (map drawing and picture classification task, probing route and survey knowledge). This pattern of age differences in the wayfinding and spatial memory performances not only fully agrees with our previous study focusing on the virtual-real transfer of spatial learning in order to complete routes in the real environment (21], but also is in accordance with the study by Foreman et al. [30]. Therefore, this is consistent with our previous interpretation that the virtual-real transfer procedure means that the elderly adults have better spatial learning through the *re-test* effect, notably during the wayfinding task. An additional explanation is that the use of a virtual environment that is based on a real environment to perform a transfer task may enhance spatial learning in older adults because these VEs closely match the physical characteristics of real environments. Older adults have many years of experience with regards to spatial learning in unfamiliar environments, which could have contributed to the long-term formation of a generally higher level of spatial knowledge (i.e., topographical schema) compared with younger people. It appears likely that the use of virtual environments based on real environments, compared to artificial environments, provides more environmental support [65], and thus activates this topographical knowledge. Subsequently, this enables the elderly adults to be more efficient in their spatial learning or even, to benefit more from the re-test effect (also see [66] for a similar interpretation of spared navigation performances in elderly persons for everyday tasks). In any case, the present results indicated that the spatial learning difficulties experienced with aging can be compensated for, whereas the learning difficulties observed for wayfinding are more robust, given the more critical role played by executive functioning in this task.

Second, it is interesting to note that for both the younger and the older participants, a detrimental effect of active learning with the joystick was observed on the map drawing task (probing the survey level) while no significant effect was observed on the picture classification task (probing the route level). This indicated that, under the active condition, motor control of a joystick worsened spatial learning, specifically in the acquisition of elaborate survey knowledge. With regards to the joystick-based active condition, similar results have been reported by Sandamas and Foreman [44]; however, other studies also report different results (e.g., *positive effect*: [36], [37], [38], [67]; *null effects*: [68]). As stressed by Chrastil and Warren [4] and Sandamas and Foreman [44], among the main reasons for the inconsistent effects of a joystick condition, the impoverishment of motor processing as well as a less automatic mapping between motor efference and visual reafference for movement control (compared to a real legged locomotion) probably play a critical role in the resulting effect on survey knowledge acquisition, particularly for large-size environments. Indeed, passive participants could certainly focus on viewing and learning the environment layout while the active participants’ efforts were divided between operating the input device, implementing directional instructions while simultaneously learning the task.

By contrast, motor control has a less deleterious effect in the acquisition of route knowledge. This is consistent with previous results showing that the manipulation of intentional/incidental encoding of route knowledge does not have an impact on the identification of landmarks and putting those landmarks in the correct temporal order [15]. Nevertheless, as highlighted by Chrastil and Warren [4], if some aspects of route knowledge acquisition can be as cognitively effortless as the temporal order of landmarks, some others, such as place-action association or route reproduction can be more effortful like the acquisition of survey knowledge. Similarly, idiothetic processing (locomotor efference, proprioception and vestibular information afforded by walking activity), which varies in resource demands, is sometimes demonstrated as promoting survey knowledge acquisition (e.g., [17] and for a review, see [4]). Thus, an extended measurement on all of the facets of route knowledge acquisition as well as those of survey knowledge acquisition might capture some differential effects of joystick-based active learning as a function of their respective resource demanding. Further studies are necessary for a better understanding of the influence of motor activity relative to joystick manipulation on spatial learning in VR devices.

In any case, if the survey knowledge acquisition measures are more dependent on effortful processes compared to the measures for route knowledge [4], [10], [11], [12], [13], the selective detrimental effect of motor control of a joystick can be seen as a dual task effect due to resource competition on the survey task. This conclusive result may give insights into the inconsistent findings reported in the studies on younger adults, relative to the active exploration effect, according to the different levels of spatial knowledge. Indeed, as the competition for processing resources within a dual-task depends on the allocation resource initially required for each of the two tasks [69], inconsistent findings such as null or positive active exploration effects can likely reflect spatial learning tasks that are not very resource demanding. For instance, the size of the virtual environment used is often relatively small (one building with one level and a few hallways), and therefore the survey representation acquired is not complex and probably not a resource-limited task. Hence, it is likely that the addition of the motor control condition is not critical (null effect) or, even more likely, it could be a source of self-involvement in the task for the participant, thus explaining some positive effects reported in several studies. In fact, some studies found a positive effect of motor activity on the self-involvement of the participants [70], [71].

In all cases, the present results revealed the importance of assessing spatial learning abilities with virtual environments that are sufficiently large and as rich as real life environments in order to capture a dual-task effect (due to motor activity) in the subjects’ spatial learning performance. In a more general manner, this means that the effects of motor activity are strongly dependent on the relationships between cognitive and motor processing during spatial learning.

Finally, and most importantly, we found a positive effect of active learning in the two wayfinding performance scores for the younger group [14], [33], [34], [35], whereas a negative effect in these two scores was exhibited by the older group. Accordingly, as expected, an active virtual exploration of the virtual district enhances the subsequent wayfinding performances in the real condition of the younger subjects, but is detrimental for the elderly participants. This aging effect mirrors the well known increase of age-related differences in dual-task situations [72], notably in competitive situations between “cognitive control” and “motor control”, such as walking and talking or memorizing [47]. This interpretation is supported by correlation results showing that when the mediating effects of executive functioning are controlled, it considerably reduces the relationship between age and the wayfinding score for the active condition. Interestingly, in the passive condition, the correlation between age and the wayfinding performances was no longer significant when each one neurocognitive scores were controlled. This latter result is consistent with our previous results which indicated that the wayfinding difficulties observed in the virtual-real transfer procedures originate from age-related failures in the complex orchestration of multiple cognitive processes required to perform the wayfinding task ([21]; for a review, see [3]). Thus, while wayfinding difficulties are related to multiple facets of aging decline under the passive condition, they are broadly related to executive decline under the active condition as detrimental consequence of sensorimotor activity simultaneously performed with a spatial learning task.

Considered together, and compared to the passive condition, the results indicate that the age-related differences in the wayfinding performances under the active condition are overall related to an age-related decline in executive processes. This corroborated our hypothesis that the addition of motor control during spatial learning can be seen as a dual-task condition that in turn, requires the mobilization of more resource demanding processes to subsequently perform wayfinding tasks. This age-related dual task effect was also found in a similar study to ours, in which participants had to walk on a treadmill and learn the configuration of a virtual museum [28]. Two conditions were compared: the participants were allowed to control their balance with hand trails in the first condition but not in the second. These two conditions showed comparable performances in the younger participants. On the contrary, the condition in which participants had to control their balance themselves induced worse spatial memory performances in the older group. According to the sensory deficit theory [49], this age-related dual task effect was explained by the sensorimotor decline for balance control [73], which in turn requires higher working memory resources and finally affects simultaneous cognitive activities. Thus, the effect of the age-related dual task reported here can be seen as a sensorimotor deficit for joystick use that induces an additional cognitive load for the elderly participants during the spatial learning phase.

Although a dual-task effect interpretation of motor activity during learning can explain the older subjects’ performances, this is less the case for the younger subjects’ performances. Despite an actual detrimental effect of the active condition on the survey knowledge score, the younger adults paradoxically exhibited better wayfinding performances after the active learning condition than after the passive learning condition. This apparent paradox might be due to the fact that survey knowledge was not necessary for the wayfinding task as sometimes reported in studies [20]. Therefore, one explanation of young subjects’ benefit from the active condition may be that the younger adults probably adopt a route knowledge strategy based on the visual recognition of landmarks and route sequences to infer the correct path. This view is supported by the fact that an active condition worsened the survey knowledge performance but not the route knowledge performance (i.e., picture classification task). Furthermore, a wayfinding strategy based on route knowledge is likely to be much more useful in a wayfinding task that is based on path recognition from a virtual to a real condition according to the well-known transfer-appropriate processing effect in the memory domain [74]. The elderly adults may have flexibility difficulties to implement a wayfinding strategy (i.e., to prioritize a route strategy over survey or mixed survey-route strategy, as done by the younger adults). This interpretation is also proposed by Etchamendy et al. [75], who showed that when older adults have not acquired survey knowledge of the environment they are more inclined to exhibit worsened wayfinding difficulties in virtual maze tasks. This lack of flexibility to use an optimal wayfinding strategy may also be related to less awareness of wayfinding difficulties in everyday life. Even if further evidence should support our interpretation, it could be assumed that the age-related paradoxical effect of active learning in wayfinding performances is related to an executive decline with aging in terms of a wayfinding coping strategy based on the available spatial knowledge.

To conclude, as a dual-task effect, this study is the first to provide behavioral-based evidence showing that motor activity during spatial learning reduces the acquisition of survey knowledge. The consequence of this is an increase in the age-related differences in the wayfinding performances primarily associated with the executive factors. These findings thus stress the critical role of motor interfaces that must be taken into account in future aging studies that are based on VR devices.
